# Variations in Wettability and Interfacial Tension during Alkali–Polymer Application for High and Low TAN Oils

**DOI:** 10.3390/polym12102241

**Published:** 2020-09-29

**Authors:** Vladislav Arekhov, Rafael E. Hincapie, Torsten Clemens, Muhammad Tahir

**Affiliations:** 1 OMV Russia Upstream GmbH, 197046 St. Petersburg, Russia; Vladislav.Arekhov@omv.com; 2 OMV Expl. & Production GmbH, New Technologies, Trabrennstraße 6-8, 1020 Vienna, Austria; Torsten.Clemens@omv.com; 3 Institute of Subsurface Energy Systems, Clausthal University of Technology, 38678 Clausthal-Zellerfeld, Germany; muhammad.tahir@tu-clausthal.de

**Keywords:** EOR, interfacial tension, recovery factor, polymer flooding

## Abstract

The injection of chemicals into sandstones can lead to alterations in wettability, where oil characteristics such as the TAN (total acid number) may determine the wetting state of the reservoir. By combining the spontaneous imbibition principle and the evaluation of interfacial tension index, we propose a workflow and comprehensive assessment to evaluate the wettability alteration and interfacial tension (IFT) when injecting chemical-enhanced oil-recovery (EOR) agents. This study examines the effects on wettability alteration due to the application of alkaline and polymer solutions (separately) and the combined alkali–polymer solution. The evaluation focused on comparing the effects of chemical agent injections on wettability and IFT due to core aging (non-aged, water-wet and aged, and neutral to oil-wet), brine composition (mono vs. divalent ions); core mineralogy (~2.5% and ~10% clay), and crude oil type (low and high TAN). Amott experiments were performed on cleaned water-wet core plugs as well as on samples with a restored oil-wet state. IFT experiments were compared for a duration of 300 min. Data were gathered from 48 Amott imbibition experiments with duplicates. The IFT and baselines were defined in each case for brine, polymer, and alkali for each set of experiments. When focusing on the TAN and aging effects, it was observed that in all cases, the early time production was slower and the final oil recovery was longer when compared to the values for non-aged core plugs. These data confirm the change in rock surface wettability towards a more oil-wet state after aging and reverse the wettability alteration due to chemical injections. Furthermore, the application of alkali with high TAN oil resulted in a low equilibrium IFT. By contrast, alkali alone failed to mobilize trapped low TAN oil but caused wettability alteration and a neutral–wet state of the aged core plugs. For the brine composition, the presence of divalent ions promoted water-wetness of the non-aged core plugs and oil-wetness of the aged core plugs. Divalent ions act as bridges between the mineral surface and polar compound of the in situ created surfactant, thereby accelerating wettability alteration. Finally, for mineralogy effects, the high clay content core plugs were shown to be more oil-wet even without aging. Following aging, a strongly oil-wet behavior was exhibited. The alkali–polymer is demonstrated to be efficient in the wettability alteration of oil-wet core plugs towards a water-wet state.

## 1. Introduction

Enhanced oil-recovery (EOR) methods affect reservoir rock and/or reservoir fluids to increase the displacement and sweep efficiency of oil [1,2]. Different EOR methods are described in the literature, focusing, for instance, on chemical methods (e.g., involving polymers and alkali materials) and more specifically, on wettability alteration during alkali–polymer flooding.

A set of laboratory methods is available to define the wettability of core plugs, e.g., contact angle measurements, the Amott–Harvey method, and the U.S. Bureau of Mines (USBM) method [3]. One of the most well-known and widely used methods to deduce or assess wettability is the Amott imbibition test, which is applied not only to sandstones but also carbonates [4,5]. Amott spontaneous imbibition tests have been widely used in the industry for wettability evaluation [6]. The imbibition process is governed by the capillary suction phenomenon which, in turn, is directly related to the wetting state of the system and the interfacial tension (IFT) between fluids [7]. Hence, measuring both parameters (wettability and IFT) can help determine the relative changes in wettability following the application of chemicals. Selected works from the literature are summarized in Table 1.

The role of interfacial tension (IFT) in wettability evaluation has been widely reported in the literature (e.g., Schechter et al. [17], Sharma et al. [18]). To recover residual oil, studies reported by Skripkin et al. [19], Tong et al. [20], and Arihara et al. [21] have shown that IFT should be reduced to ultra-low values in the order of 10^−3^ N/m. Under low IFT, irremovable oil with large forces at the interface is turned into movable oil that allows easy deformation [22]. Furthermore, it was demonstrated that transient IFT plays a larger role than equilibrium IFT in the release of residual oil [20]. A rapid change in local IFT might induce deformation of the interfacial films and, combined with the help of fluid-driven shear forces, the residual oil will start moving forward [23].

Many laboratory investigations have been performed to identify alkali-flooding effects on incremental oil recovery. Various authors (e.g., Ehrlich et al., [22]; Emery et al., [24]; Skripkin et al., [19]) have reported the effectiveness of using alkali solutions for flooding purposes to increase oil production. In addition, full field studies of alkali, alkali–polymer, and alkali–surfactant–polymer (ASP) injections have provided promising results. For instance, as reported by Yuan et al. [25], the ASP flooding implementation in China’s oil fields has shown an average 15% increase in oil recovery. Skripkin et al. [19] also reported a 38% increase in oil recovery via ASP flooding at the White Castle field. A field pilot test was performed by Shell in 1989 in several wells. Additional oil production during alkaline flooding is attributed to two main mechanisms: the low IFT between fluids and the wettability alteration [5,25]. Both effects promote an increase in the capillary number, leading to incremental oil recovery. The reduction in interfacial tension with the addition of an alkali solution mainly occurs due to in situ soap generation mechanisms. The composition of crude oil plays an important role during alkaline flooding. Its efficiency mainly depends on the amount of natural acids in the oil, which can be neutralized by alkali. This finally leads to in situ soap creation and, consequently, to a decrease in IFT [20,26,27]. A number of research projects have focused on confirming IFT reduction during alkali application.

Several different parameters can be used to describe or determine the potential for alkali flooding, such as the total acid number (TAN) of crude oil as measured using titration techniques. Although recent studies could not find a correlation between incremental oil recovery due to alkali application and TAN, Sheng [28] considers the effect of this correlation to be indisputable. Furthermore, it is known that the brine composition and mineralogy of a sample determine the extent of wettability alteration [3,29,30,31].

In this work, we investigate the wettability alteration and interfacial tension changes due to the application of alkali–polymer. This investigation was performed by varying different parameters with a focus on (1) the total acid number of the oil; (2) the influence of the aging of the core plugs; (3) changes in brine composition, and (4) variations in mineralogy (clay%).

Note that, oil, brine composition and, core plugs were chosen to mimic rock-fluid properties of the 16 TH reservoir of Matzen field (Austria), where the field-scale alkali-polymer application is under consideration, as described by Schumi et al. [16].

The paper is organized into four main sections: First, the overall methodology and approaches are described, follow by a detailed description of data for the fluids and core samples in the second section. Third, the methods for IFT and Amott spontaneous imbibition are described. The results are subsequently presented and discussed based on defined cases. Finally, we draw conclusions regarding the observations.

## 2. Materials and Methods 

### 2.1. Methodology and Approach

Here, we present a methodology to evaluate and further determine the wettability changes and interfacial tension reduction when applying alkali–polymer flooding using high and low TAN oils. For this process, the following workflow was adopted:

1. Phase experiments to define chemical blend concentrations. Leitenmüller et al. [32], Schumi et al. [16], and Neubauer et al. [33] reported these experiments.
2. The selection and characterization of brines that differ in ion content to define the influence of monovalent and divalent ions on IFT and wettability. According to Awolayo et al. [30] and Mohnot et al. [31], brine composition can determine the extent of wettability alteration.
3. For a potential field pilot, we chose to use oil from a defined reservoir; hence, oils with high and low TAN values were selected and further studied.
4. Interfacial tension was measured as a function of time over 300 min at a temperature of 60 °C for the defined chemicals and oils. The evaluation was based on the assessment proposed by Sharma et al. [18].
5. Sandstone core plugs that clearly differ in clay content (2.5% and 10%) were selected, and routine core analysis was performed. Both outcrops resemble the permeability of the envisaged reservoir. The mineralogy of the sample may determine the extent of wettability alteration [3,29].
6. Core plugs were saturated to determine irreducible water (Swirr) and initial oil saturation (Soi). Permeability to water and oil were defined during the saturation process and cross-analyzed.
7. Two groups of cores were considered during the process, aged and non-aged, to assess any wettability shift at reservoir temperature. Core aging at reservoir temperature for around 800 h was reported to lead to a wettability shift from a purely water-wet state of the cleaned core samples to a neutral or oil wet state [3,34,35].
8. Amott spontaneous imbibition experiments were performed for different defined cases considering the influence of TAN, core plug aging, brine composition, and clay rock content. The results were assessed by a detailed analysis of oil production with time dependency and final oil recovery.

### 2.2. Fluids and Core Sample Data

#### 2.2.1. Brines

Two synthetic brines were prepared to investigate the influence of brine composition on the spontaneous imbibition process and IFT. First, a simplified brine was used without divalent cations, which is named here as test water. This simplified brine was used to mimic the salinity and buffering capacity (e.g., pH and alkalinity) of the reservoir water. Note that, the buffering capacity (NaHCO_3_) helped having a controlled pH environment. Nevertheless, as reported by Schumi et al. [16] the pH value was controlled between 9.6 to 9.8. Second, a brine with similar composition to that of the 16 TH reservoir was prepared (formation brine). The composition and main parameters of the brines can be seen in Table 2.

As background information, this study complements previous studies performed for similar purposes. Previous studies performed by Leitenmüller et al. [32], Schumi et al. [16], and Neubauer et al. showed an in-depth investigation on the possible effect of pH for the used alkali–polymer solutions here presented.

#### 2.2.2. Oil Samples

Two types of crude oil were used in this work: one from the 16 TH reservoir of Matzen field (Austria, well Bo-112) and another from the St. Ulrich reservoir. On the one hand, the 16 TH oil was moderately degraded oil with a composition characterized by 39% saturated compounds, 20% aromatic compounds, and 39% bearing compounds such as nitrogen, sulfur, and oxygen, with the asphaltene content being ~2% [36]. On the other hand, the St. Ulrich oil was characterized by 6% polar compounds, 17% aromatics, 76% paraffin compounds, and asphaltene content of <1% [37]. Both crude oils were chosen for the comparison of their behavior based on the TAN (total acid number). The 16 TH crude oil possesses a high TAN, whereas the St. Ulrich crude oil has only a small amount of acid (Table 3). Although it is already known that a high acid value is generally preferred for alkali flooding, the main reason for comparing high and low TAN oil is to better capture the differences or influences—if any—of low TAN oils.

#### 2.2.3. Chemical Solutions

Chemicals selection and the optimization and definition of the concentrations were previously performed and reported by Schumi et al. [16]; subsequently, the phase behavior experiments were complemented by Neubauer et al. [33]. The evaluation was performed based on phase behavior, micro-model, and coreflooding experiments, where an optimum alkali and polymer concentration and the type of alkali and polymer to be used were defined. Sodium carbonate Na_2_CO_3_ at a concentration of 7000 ppm was considered as the alkali solution, and FLOPAAM 3630S (a hydrolyzed polyacrylamide, HPAM polymer) from SNF Floerger was dissolved in the specific brine at a concentration of 2000 ppm. The alkali–polymer solution was defined as a combination of its constituent solutions (concentrations). The main properties of the chemical solutions can be found in Table 4.

#### 2.2.4. Core Samples

**Figure 1:**
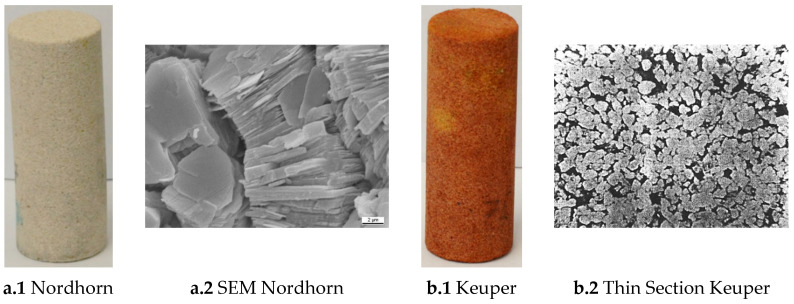
Images of the outcrop core used in this work: Nordhorn (**a.1** and **a.2**) and Keuper (**b.1** and **b.2**). SEM refers to scanning electron microscope.

During the investigation, a total of 48 core samples were used (40 Nordhorn, a Bentheimer-like outcrop, and 8 Keuper core plugs). Two different rock types were considered to study the effect of mineralogy during the imbibition process. Nordhorn (NH) Core Figure 1a shows a similar Bentheimer-like outcrop rock from Germany. The sample can be described as fine–medium-grained porous sandstone that consists of quartz (96.6%), potassium feldspar (0.9%), and kaolinite (2.5%). Due to its mineralogical composition, the sandstone can be referred to as quartz arenite weakly cemented by quartz and clay materials. The quartz grains exhibit well-developed quartz cement overgrowths (see Figure 1a). Nordhorn was chosen for the following reasons:

1. The permeability of the core plugs resemble the permeability ranges of the 16 TH formation where alkali–polymer flooding is under investigation for possible implementation [16].
2. The core plugs are highly similar in their properties (their observed ranges and differences in porosity and permeability values are narrow).
3. The rock mineralogy is simple, thereby simplifying any additional uncertainties during the experimental analysis and facilitating data acquisition.

#### 2.2.5. Keuper Core

Keuper is an outcrop from Germany and was selected due to its high heterogeneity and high content of clay material. These features enable the observation of the possible influence of clay particles on the wettability alteration process (see Figure 1b). Keuper is a reddish-brownish fine-grained sandstone and black spots can be observed in some specific areas. Thin-section analysis revealed that the sample is a well-sorted sandstone arenite composed of quartz (88%), feldspar (1%), kaolinite (10%), and other materials (1%) (see Figure 1b). The porous space is predominantly primary interparticle porosity. Only a minor part of the porous space originated due to secondary porosity in the feldspar.

**Figure 2:**
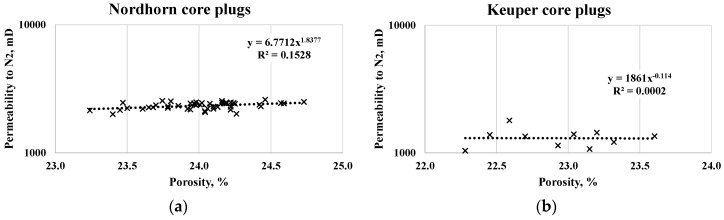
Porosity-N_2_ permeability cross-plot for the Nordhorn (**a**) and Keuper core plugs (**b**).

A summary of the routine core analysis (RCA) and further saturation data are listed in Table 5. Measuring the permeability to gas (N_2_) provided consistent data when comparing the porosity–permeability correlation (see Figure 2). The average permeability of the Nordhorn core plugs was 2313 mD, with a ±7% standard deviation, whereas for the Keuper cores, this value was significantly lower with a higher standard deviation of 1327 mD ± 24%. This again highlights the highest level of heterogeneity in the Keuper core plugs and the similarity of Nordhorn cores. Note that although the log scale ranging between 1000 and 10,000 suggests large variability, the values obtained for each core set were within the range of acceptable error. Gas permeability was measured in single-phase flow and corrected by Klinkenberg effects, while the water permeability (using test water) was defined prior to oil saturation in a single-phase flow.

**Figure 3:**
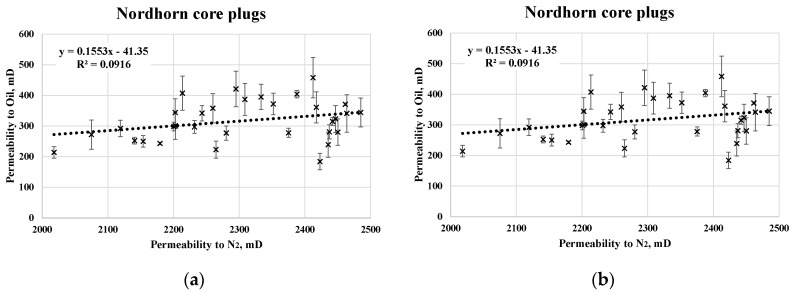
Cross-plot of permeability to gas and oil without outliers for the Nordhorn (**a**) and Keuper samples (**b**).

A summary of the relevant core data and mean values of two-phase analysis is presented in Table 5. The results obtained from the RCA indicate a reasonable standard deviation. A large number of core plugs were analyzed, which boosts confidence in the reliability of the data. When referring to saturation data, the irreducible water saturation in the Keuper cores was significantly larger than that in the Nordhorn core plugs. This could be the result of a more complex porosity distribution within the core plug. Despite differences in the irreducible water saturation values, the end-point relative permeability levels were shown to be similar. Note that oil saturation was assessed at 5 different rates to ensure uniform saturation and minimize the capillary end effect. Rates were chosen to avoid the destruction of pore throats. An example of the oil permeability evaluations is shown in Figure 3. Good correlation was observed between the permeability to oil and N_2_.

### 2.3. Interfacial Tension (IFT) Evaluations

For interfacial tension, a KRUSS spinning drop tensiometer was used. The heavy fluid (e.g., brine, brine + alkali + polymer) was placed into the vessel using a piston and a rod. A droplet of oil was used to fill an oil holder, which was then fixed onto a vessel. IFT was measured at 60 °C and 7000 rpm based on the principles suggested by Sharma et al. [18]. Each solution was measured two to three times until the standard deviation was fairly low to define/ensure a low uncertainty range. Notably, the obtained results indicate the complexity of measuring IFT and the high associated uncertainty, especially for IFT systems with a >1 mN/m range. For the 16 TH oil, Arnold [38] gathered similar observations. Although the exact values of the measured IFTs might not be strictly representative, they provide enough information for fluid comparison based on IFT indicators. 

Overall Observations for IFT Data Interpretation 

The changes in IFT were recorded and observed over 300 min, revealing both instant IFT values (right after the start of the experiment) and the equilibrium period for IFT (expected to be established after the reaction between fluids). The observed standard errors during the experiments were possibly due to the following reasons:

1. Different algorithms considered for the measurement based on drop shape and fluid characteristics.
  Two different algorithms were available for the measurements (Vonnegut and Young–Laplace). For systems with an interfacial tension greater than 1 mN/m (test water and polymer solutions), the Vonnegut algorithm could not be applied because of the oil droplet shape. Therefore, the Young–Laplace algorithm was used. At the same time, for systems with an IFT lower than 1 mN/m (alkali and alkali–polymer solutions), the Young–Laplace method could not be used because of the highly elongated shape of the oil droplet. Hence, the Vonnegut method was implemented in these cases.
2. Lack of proper temperature stabilization leading to a malfunction of the device.
  From time to time, the recording camera stopped responding, which led to missing data for this period until the camera was set up again.
3. The collapse and/or conjunction of multiple oil droplets.
  Some errors due to collision and separation of multiple oil droplets were observed. In the case of low IFT (alkali and alkali–polymer solutions), the droplets were especially unstable over time. Due to separation/collision of the droplets, the volume changed and, consequently, the IFT measurement was affected.

### 2.4. Contact Angle Measurements

Contact angle measurements were performed using a high pressure view cell (HPVC) 40-MD (Novis DN15, PN40; maximum operating conditions (Pmax/Tmax): 40 bar/200 °C). A piston accumulator was connected to the inlet of the cell for the injection of a heavy fluid “alkali–polymer/alkali/brine/polymer” and pressure exertion. On the bottom of the view cell, a capillary that dispenses oil was connected (16 TH/St.Ulrich) for the analysis of the contact angle. The cell was exposed to a light source to enable camera recording on the other side of the cell for taking high-contrast images. The temperature inside the view cell was controlled with a fluid-circulated heating jacket. The fluid temperature was monitored using a thermocouple (thermocouple type K with resolution of 0.1 K and accuracy of ±1 K).

**Figure 4:**
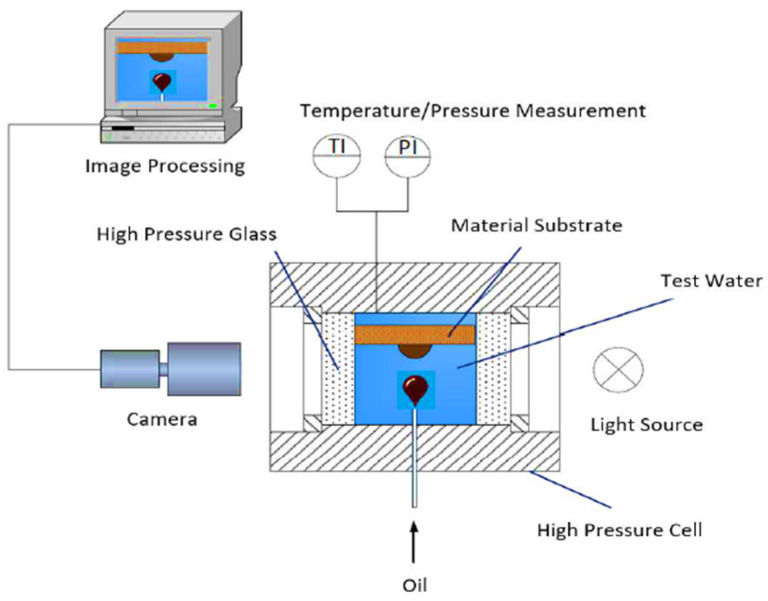
A high pressure and temperature KRUSS spinning drop tensiometer high pressure view cell (HPVC) 40-MD for contact angle measurements.

A graphical representation of the experimental setup is included in Figure 4. Contact angle measurements were performed prior to the aging process of both core sample types, with some additional points measured after. Oil drops of both oil samples (mentioned in Table 3) were produced in various aqueous phases (brine, alkali, polymer, and alkali–polymer).

### 2.5. Spontaneous Imbibition Evaluations

**Figure 5:**
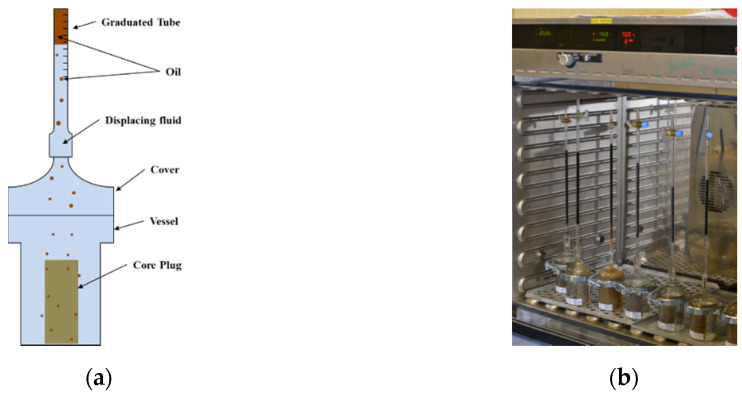
Schematic representation of the Amott imbibition cell (**a**) and the set experiments placed in the oven (**b**).

Amott cells were used for spontaneous imbibition experiments. The design of the cell is simple and consists of a vessel where the sample is placed and covered by a top with a graduated tube for oil production recording. A schematic representation of the Amott cell is shown in Figure 5. The experimental principle is straightforward: the core saturated with oil until irreducible water saturation is submerged into the displacing fluid. The oil production over time is recorded by measuring the volume in the graduated pipette. To replicate temperature reservoir conditions, Amott cells were placed in the oven at a 60 °C temperature.

**Figure 6:**
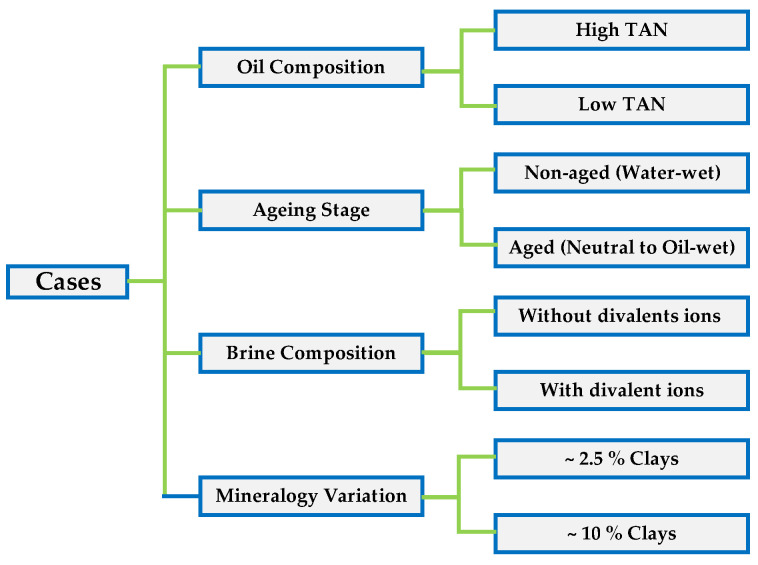
Cases/scenarios and parameters used to understand the data gathered from the Amott spontaneous imbibition tests.

To better structure the evaluation and gathering of data, we set different scenarios which allowed us to cross-analyze and understand the effects of each parameter: (1) total acid number of the oil; (2) the influence of core plug aging; (3) changes in brine composition; and (4) variation in mineralogy (clay%). A schematic representation of the cases is shown in Figure 6. Experiments were conducted a minimum of two times to assess the uncertainty range related to the test procedure. The error bars shown in the diagrams represent the measurement uncertainties.

According to the defined cases, the behavior of oil production due to imbibition was studied based on two factors:

1. Oil production with time dependency (revealing how capillary forces will perform over time);
2. Final oil recovery, which represents the total amount of oil trapped in the system.

Note that the Amott imbibition test can potentially only provide a partial picture of wettability compared to the complete overview given by Amott–Harvey wettability index or USBM, in which spontaneous and forced drainage and imbibition are performed. Force evaluations and interpretations are presented in Schumi et al. [16].

## 3. Results and Discussion

### 3.1. Interfacial Tension Observations

**Figure 7:**
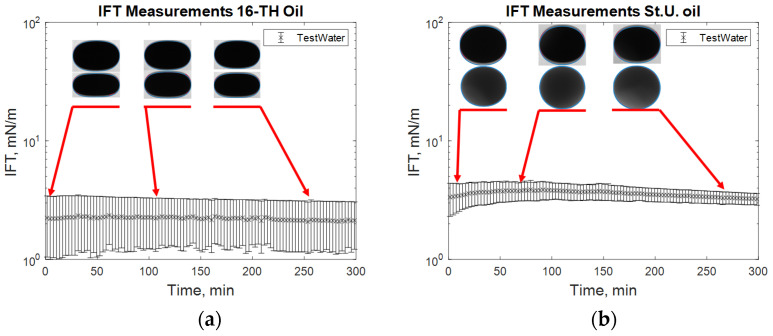
Interfacial tension (IFT) measurements for high total acid number (TAN) (**a**) and low TAN oil (**b**) with test water at 60 °C.

**Figure 8:**
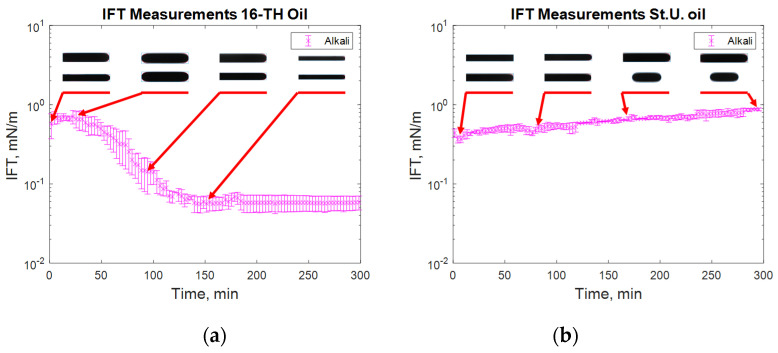
IFT measurement for high TAN (**a**) and low TAN oil (**b**) with the alkali solution at 60 °C (alkali: 7 g/L Na_2_CO_3_).

**Figure 9:**
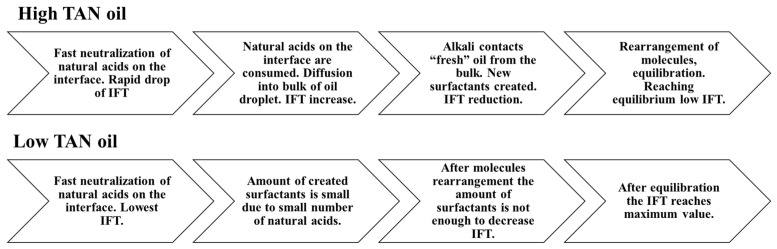
Schematics explaining the process of IFT change for high and low TAN oils_._

**Figure 10:**
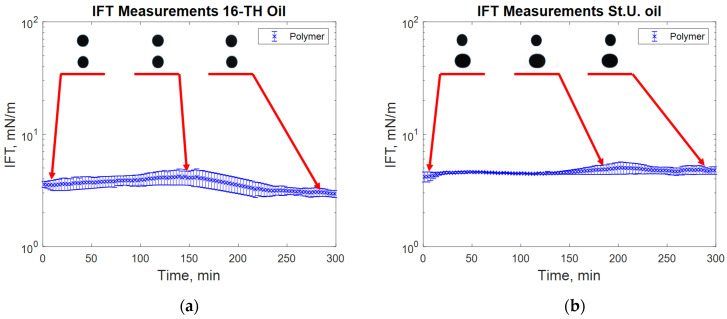
IFT measurement for high TAN (**a**) and low TAN oil (**b**) with the polymer (P) at 60 °C (polymer: 2000 ppm FLOPAAM 3630S).

**Figure 11:**
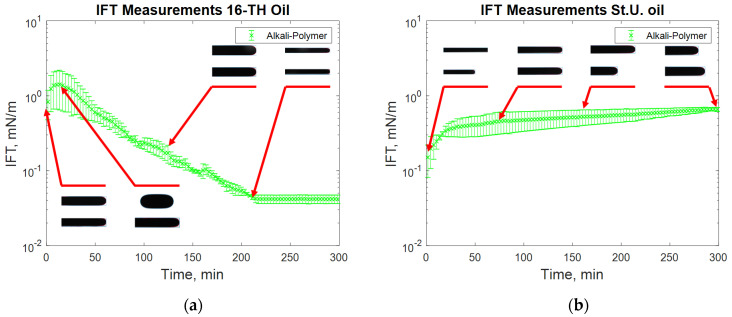
IFT measurement for high TAN (**a**) and low TAN oil (**b**) with the alkali–polymer at 60 °C (alkali: 7 g/L Na_2_CO_3_; polymer: 2000 ppm FLOPAAM 3630S).

The reported data are in good agreement with those previously reported by Leitenmüller et al. [32], Schumi et al. [16], and Neubauer et al. [33], considering that all these data were measured in different periods. Each of the experiments (the tested kinds) is described in detail using images of the droplet sizes and the measured IFT over time during the same periods (see from Figure 7, Figure 8, Figure 9, Figure 10 and Figure 11). In Figure 7, it can be observed that in the case of both high and low TAN oils, the effect of alkali on lowering interfacial tension is clear. However, the decrease in IFT for high TAN oil is more significant, and the final equilibrium value reaches a low IFT in the range of 0.05 mN/m. For all cases, the measured values of interfacial tension between the oils and test water stay constant over time, ranging between 1 and 3.5 mN/m for high TAN oil and 2 to 4.5 mN/m for low TAN oil.

The addition of a polymer caused a slight increase in the measured IFT values (Figure 7), whereas the highest reduction in IFT was caused by the alkali’s interaction with high TAN oil (Figure 8). The application of the alkali together with the polymer resulted in slightly higher interfacial tension values (Figure 8). Presumably, the higher interfacial tension observed for the alkali–polymer compared to the pure alkali solution indicates a lower concentration of alkali (due to the additional volume of the polymer) and a slower diffusion of Na_2_CO_3_ molecules into the oil due to higher polymer solution viscosity. Another relevant observation is the low instantaneous IFT observed for the case of the alkali and alkali–polymer solution with high TAN oil. As per repeated experiments, the oil droplet elongated drastically within the first minutes of the measuring period (right after the start of the measurement), followed by a shrinking period, again delivering a higher value of IFT. Similar behavior was observed during the spontaneous imbibition evaluations, which will be discussed further.

Figure 7 shows the behavior of the oil droplet during the IFT measurements with test water for both oils. The droplet shape remains almost constant over the 300 min of the experimental period. Only a slight increase in IFT was observed at approximately minute 50 for the experiment with low TAN oil. Comparing the repeated measurements for the same solution helped in clarifying the effect of the droplet size (oil) on the IFT value outcome. During the second measurement for high TAN oil, the oil droplet with a smaller volume was formed. This led to lower measured values of the IFT and resulted in a high range of uncertainty for the experiment.

For alkali solutions (Figure 8), very different behavior was observed for both high and low TAN oil. First, the instantaneous IFT for the high TAN oil reached very low values. As soon as the experiment started, the droplet quickly elongated, showing small interfacial tension. After a few minutes, the droplet started to shrink again, increasing the IFT until it reached a peak. After passing the highest value, the IFT started to decrease again, reaching equilibrium at a value of around 0.05 mN/m. Similar behavior of interfacial tension was observed by Sharma et al. [18]. Using low TAN oil led to a different outcome. The initial low value of IFT monotonically increased over the 300 min of investigation with an almost linear trend. Such results can be explained by the interactions between the alkali and natural acids of the oil.

After a very fast reaction on the surface of the oil droplet, the interfacial tension between the fluids dropped very quickly (Figure 8a). There were many anionic surfactants on the interface, which slowed alkali consumption. There were not enough natural acids on the interface to continue creation of the surfactants. Hence, the IFT increased shortly after the reaction. The observed data suggest a diffusion-dominated system in which the neutralization of organic acids occurs faster than the molecular diffusion of alkali into the bulk of the solution. However, due to molecular diffusion, when the alkali contacts “fresh” oil from the bulk, new anionic surfactants are created. By rearranging the molecules, the concentration of surfactant equalizes, leading to a decrease in IFT until reaching the equilibrium value where all the natural acids are consumed. For the case of the St. Ulrich oil (low TAN) shown in Figure 8b, the amount of natural acids was not sufficient for efficient IFT decrease. The initially created surfactants caused a decrease in IFT to its minimum value. However, due to the lack of natural acids and the small concentration of the surfactants after rearrangement, the interfacial tension started to increase again over time.

To facilitate the ease of understanding Figure 9 shows a schematic that attempts to explain the interfacial tension changes that occurred for high and low TAN oil. 

During the interfacial tension measurements with polymer solutions, no significant changes in oil droplet shape were observed, as can be seen from Figure 10. Compared to the test water, a slightly higher interfacial tension was measured. The uncertainty range of the evaluated data could be related to the chosen measurement algorithm or the devised stabilization. Nevertheless, taking the average value of IFT can offer a reliable approximation for an oil–polymer system.

Alkali–polymer solutions delivered similar results to those of alkali-only solutions (see Figure 11). The instantaneous IFT for high TAN oil was very low, which can be observed by the elongated structure of the oil droplet (Figure 11a). Subsequently, the droplet started to shrink, indicating a higher value of interfacial tension. Towards the end, the tension between the fluids decreased again because of the rearrangement of surfactant molecules in the droplet. The addition of polymer to the alkali solution led to a diminishing effect of IFT reduction and an increase in the observed IFT peak value. This might have occurred for two reasons: (1) a lower concentration of alkali due to the additional volume of the polymer and (2) the delayed diffusion of Na_2_CO_3_ molecules into the oil due to larger polymer molecules. Low TAN oil resulted in very similar behavior to the alkali–oil system. The initial low value of IFT gradually increased over time due to a lack of anionic surfactants on the interface between the oil and the second fluid.

### 3.2. Contact Angle Results

**Figure 12:**
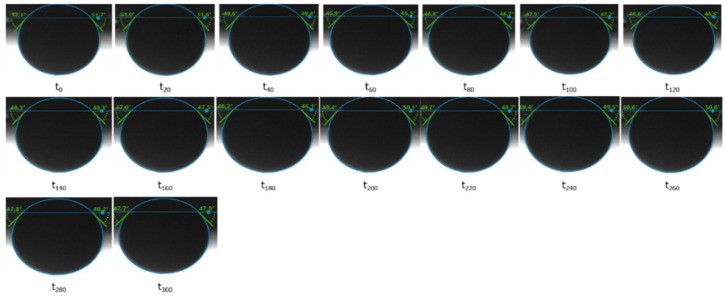
Contact angle measurements performed for the Keuper core plug in contact with the St. Ulrich oil. The alkali solution is the aqueous phase.

**Figure 13:**
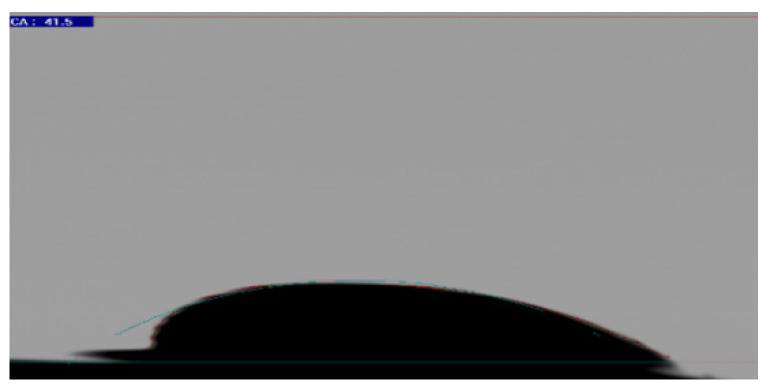
Contact angle confirming the wettability alteration to oil-wet after the aging process [34].

The contact angle results illustrated a water-wet state of both core plugs prior to the aging process, as shown in Table 6. The contact angle values were measured over time intervals of 20 min, as shown in Figure 12. As predicted based on our previous work [34,39,40,41], there was no change in the contact angle due to the strong hydrophilic characteristics of the porous media. However, the aging process altered the rock surface, which became hydrophobic, resulting in oil polar compounds attaching to the rock surface. The wettability alteration to oil-wet (shown in Figure 13), based on our previous work [35,42], confirmed the change in contact angle to 41. The wettability alteration of the Berea and Bentheimer sandstone core plugs through a two- or three-week aging process has also been established by many researchers [43,44,45,46,47]. On a similar note, we suspect that the aging process of the core plugs in this study changed the wettability to mix-wet or oil-wet, especially for the Keuper core plug with higher clay content.

### 3.3. Spontaneous Imbibition Observations

#### 3.3.1. Non-Aged Nordhorn Core Plugs—Oil Type Comparison

**Figure 14:**
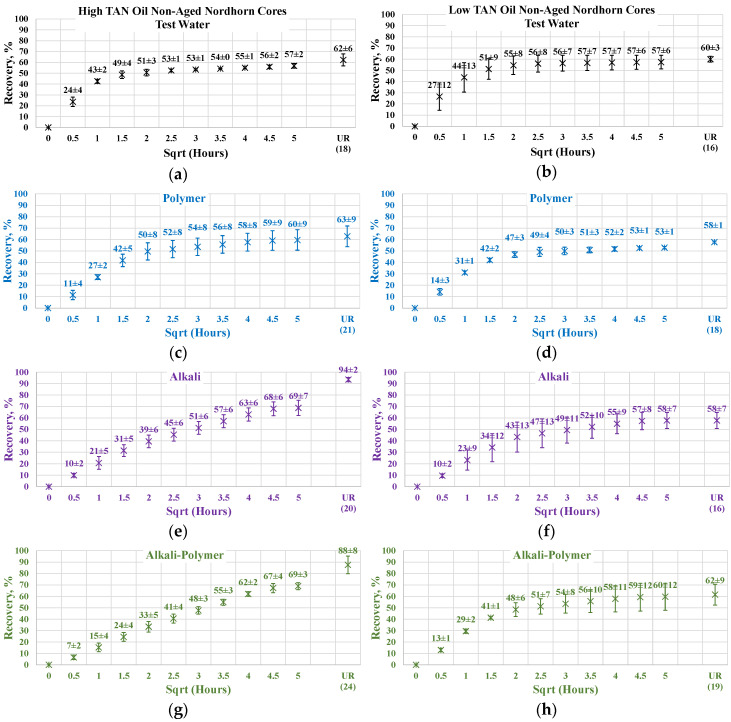
Percent of production versus the square root of the time plot for the Nordhorn non-aged core plugs using high TAN oil and low TAN oil at 60 °C (alkali: 7 g/L Na_2_CO_3_; polymer: 2000 ppm FLOPAAM 3630S). High TAN oil data (**a**,**c**,**e**,**g**) are presented on the left side whereas low TAN oil data (**b**,**d**,**f**,**h**) on the right side. Black-colored data refer to Test water, Blue-colored data refer to polymer, purple-colored data refer to alkali, and green-colored data refer to alkali-polymer. Ultimate recovery (UR) refers to recovered oil divided bv Soi.

Figure 13 illustrates the oil production behavior over the first 24 h. According to the observations in this work, this time period is the most representative. After 24 h of imbibition, only small changes in oil production were observed until ultimate recovery was reached. The ultimate recovery values for each case are presented on the bottom-right part of each graph. Note that, ultimate recovery (UR) was estimated as a volume of oil, which was recovered during the Amott imbibition test, divided by the initial volume of oil (initial oil saturation). It can be seen from Figure 14 that regardless of the TAN of the oil, the imbibition rate was larger using the test water. The observed imbibition rate for the polymer was slowed due to the large molecules in the solution. Molecules moved slowly into the pore system, which led to a lower imbibition rate. For the alkali and alkali–polymer solutions, the slower imbibition can be explained by the reduction in IFT. Due to small interfacial tension, the capillary forces were reduced, which led to a slower process of imbibition.

For the low TAN oil, all chemical solutions provided similar total recoveries with a small range of uncertainty. However, for the high TAN oil, the use of alkali and alkali–polymer solutions led to a larger amount of produced oil. Similarly, the ultimate recovery of the test water and polymer was very similar. The larger amount of total production in the case of the alkali and alkali–polymer solutions can be explained by the lower values of equilibrium IFT. Reducing the IFT to low values led to destruction of the rigid oil films, as well as the stringing and drawing of oil. Under this mechanism, the oil was produced slower, but a larger total amount was released from the core.

As shown above, low TAN oil fails to reduce IFT until ultra-low values, which is the primary reason for smaller ultimate recovery with the application of alkali and alkali-polymer solutions.

#### 3.3.2. Nordhorn Core Plugs—Influence of Aging

**Figure 15:**
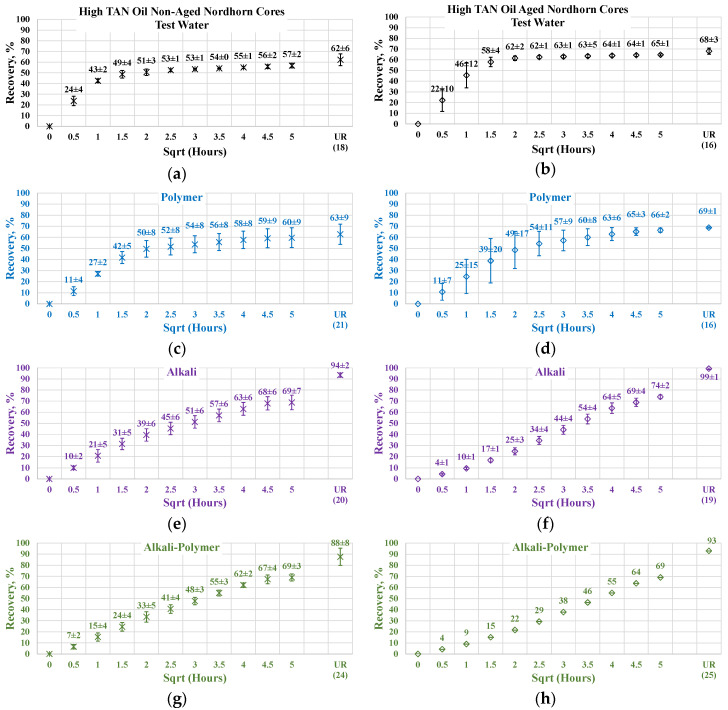
Percent of production versus the square root of the time plot for the Nordhorn non-aged and aged core plugs saturated with high TAN oil at 60 °C (alkali: 7 g/L Na_2_CO_3_; polymer: 2000 ppm FLOPAAM 3630S). Non-aged core data (**a**,**c**,**e**,**g**) are presented on the left side whereas aged core data (**b**,**d**,**f**,**h**) on the right side. Black-colored data refer to Test water, Blue-colored data refer to polymer, purple-colored data refer to alkali, and green-colored data refer to alkali-polymer. Ultimate recovery (UR) refers to recovered oil divided bv Soi.

The results of the experiments with restored wettability state core plugs are shown in Figure 15, with a comparison between the production of the aged and non-aged Nordhorn core plugs. The effect of aging was clearly deduced from the observed oil production behavior. In all cases, the early time production was slower compared to the non-aged core plugs. However, using the test water and polymer as displacing fluids produced a slower production rate only within the first hour of imbibition evaluation. All other cases exhibited significantly slower initial production rates. These data confirm the changes in the rock surface wettability towards a more oil-wet state after aging. 

The ultimate oil production outcome can be explained by the oil droplet snap-off phenomenon. Under this phenomenon, when the rock is water-wet, the invading fluid spreads around the surface, leading to a large volume of oil being left behind in the middle of the large pores. In the case of a neutral (oil)-wet state, the oil initially covers the mineral surfaces. By submerging more oil-wet core plugs into different solutions, the invading fluid enters the middle of the pore throat, releasing a larger amount of oil than in the case of the water-wet core. This explains the incremental oil recovery for all cases with the aged core plugs shown above.

The oil recovery of 80% and up to almost 100% in the case of alkali implementation suggests reverse wettability alteration from a neutral(oil)-wet state towards a water-wet state. The possible mechanism is the following. Initially, the neutral(oil)-wet core plug is submerged into the alkali solution. Fluid enters the large pores and displaces oil from the core plug. In situ-created surfactants cause wettability alteration and, consequently, changes in capillary forces. The invading fluid is then sucked into the core plug, and oil is released. Water then starts to spread around the inner surface of the pore throats. This leads to oil detachment from the mineral surfaces. Hence, the oil is released, despite being trapped in small pores and “sticking” to mineral surfaces.

**Figure 16:**
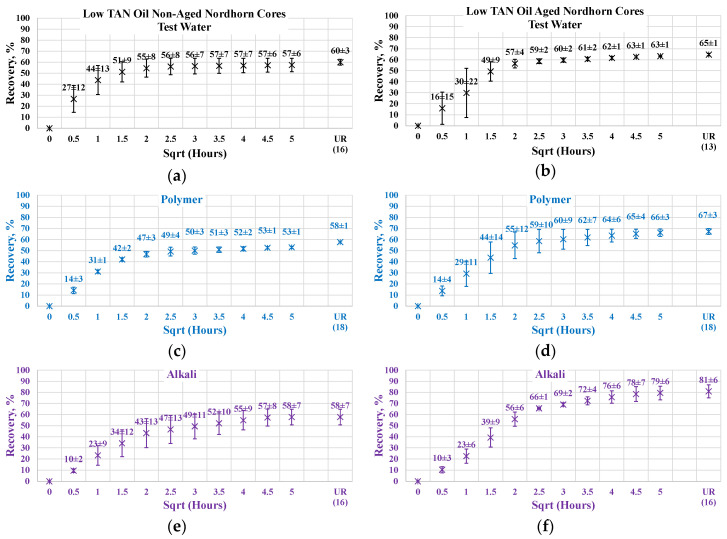

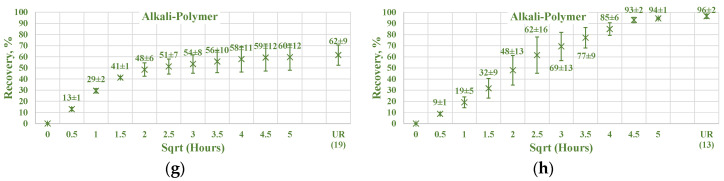
Percent of production versus the square root of the time plot for the Nordhorn non-aged and aged core plugs saturated with low TAN oil at 60 °C (alkali: 7 g/L Na_2_CO_3_; polymer: 2000 ppm FLOPAAM 3630S). Non-aged core data (**a**,**c**,**e**,**g**) are presented on the left side whereas aged core data (**b**,**d**,**f**,**h**) on the right side. Black-colored data refer to Test water, Blue-colored data refer to polymer, purple-colored data refer to alkali, and green-colored data refer to alkali-polymer. Ultimate recovery (UR) refers to recovered oil divided bv Soi.

Similar effects were observed during the low TAN oil displacement process (see Figure 16). The obtained results indicate similar behavior and confirm the previous statements. All the restored wettability core plugs exhibited slower oil production rate during the first period of the imbibition process, which further overrode non-aged core production and delivered greater ultimate recovery. As proven by the IFT measurements, even a small amount of crude oil acid was enough to decrease the interfacial tension between the oil and displacing fluid. The decrease in IFT was not large enough to cause stringing of the oil in case of a water-wet state. However, the in situ creation of surfactants resulted in wettability alteration of the initially aged core plugs. As a result, oil was released not only from the middle of the larger pores (as in the case of the test-water and polymer imbibition) but also from the mineral surfaces. This assumption was confirmed by the incremental oil production while using alkali and alkali–polymer displacing fluids for the low TAN oil case.

Nevertheless, the effect of TAN is also seen on aged core plugs. Application of alkali and alkali-polymer with high TAN oil results in higher ultimate recoveries than for non-aged core plugs, as well as for aged core plugs. A higher amount of anionic surfactants (soaps), we believe were created due to the high TAN oil affecting the wettability change to a larger extent, which is the reason for the bigger ultimate oil recovery.

#### 3.3.3. Nordhorn Core Plugs—Brine Composition

**Figure 17:**
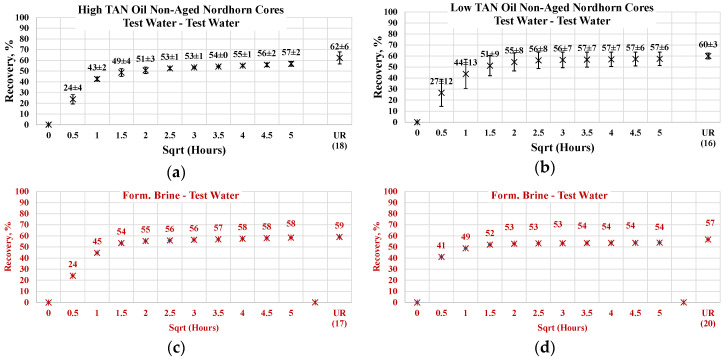

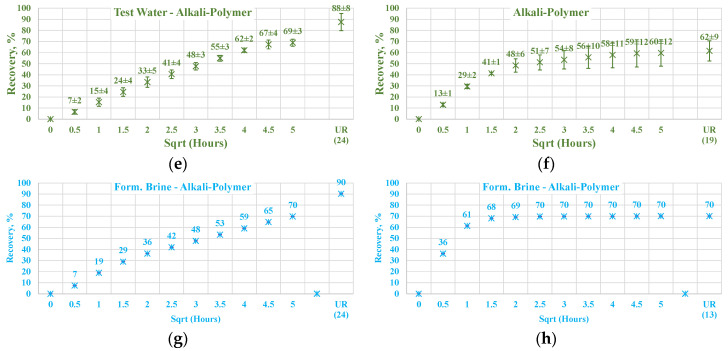
Percentage of production versus the square root of the time plot for non-aged Nordhorn core plugs (water composition comparison) for high TAN oil (**a**) and low TAN oil (**b**) at 60 °C (alkali: 7 g/L Na_2_CO_3_; polymer: 2000 ppm FLOPAAM 3630S). High TAN oil data (**a**,**c**,**e**,**g**) are presented on the left side whereas low TAN oil data (**b**,**d**,**f**,**h**) on the right side. Black-colored data refer to Test water (saturated core) imbibed by test water, red-colored data refer to formation brine (saturated core) imbibed by test water, green-colored data refer to test water (saturated core) imbibed by alkali-polymer, and blue-colored data refer to formation brine (saturated core) imbibed by alkali-polymer. Ultimate recovery (UR) refers to recovered oil divided bv Soi.

For this case, select spontaneous imbibition tests were performed with the cores initially saturated with brine containing a high amount of divalent ions. Two cases are described—one with test water and the other with the alkali–polymer solution. Figure 17 depicts a comparison of the oil production behavior in the non-aged Nordhorn core plugs for brine with only monovalent ions (test water) and one with both mono- and divalent ions (formation brine). The results indicate no significant changes in the oil production rate or ultimate oil recovery when the formation brine or test water were used for initial saturation. Only in the case of low TAN oil was the recovery higher when the core was initially saturated with formation brine and subsequently submerged into the alkali–polymer solution. This behavior can be attributed to the uncertainty while performing the measurement. As the next step, the core plugs, initially saturated with formation brine, were aged under reservoir temperatures for 30 days to determine the influence of divalent ion concentrations on wettability changes during aging.

**Figure 18:**
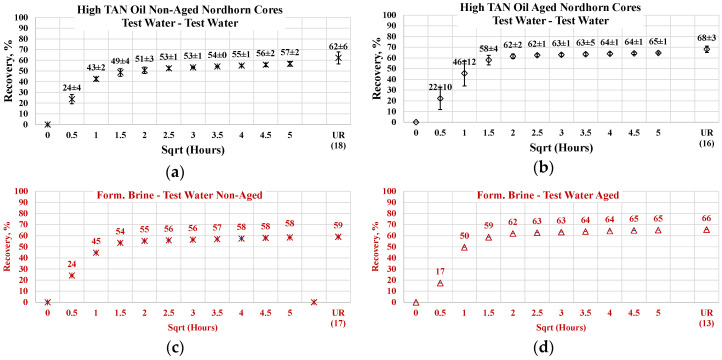

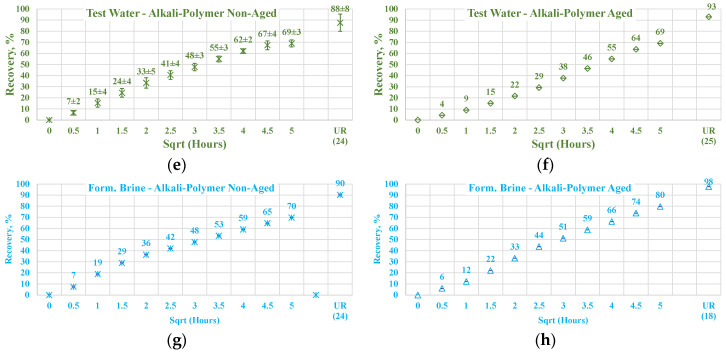
Percentage of production versus the square root of the time plot for non-aged and aged Nordhorn core plugs saturated with high TAN oil (water composition comparison) at 60 °C (alkali: 7 g/L Na_2_CO_3_; polymer: 2000 ppm FLOPAAM 3630S). Data of non-aged cores (**a**,**c**,**e**,**g**) are presented on the left side whereas aged core data (**b**,**d**,**f**,**h**) on the right side. Black-colored data refer to test water (saturated core) imbibed by test water, red-colored data refer to formation brine (saturated core) imbibed by test water, green-colored data refer to test water (saturated core) imbibed by alkali-polymer, and blue-colored data refer to formation brine (saturated core) imbibed by alkali-polymer. Ultimate recovery (UR) refers to recovered oil divided bv Soi.

**Figure 19:**
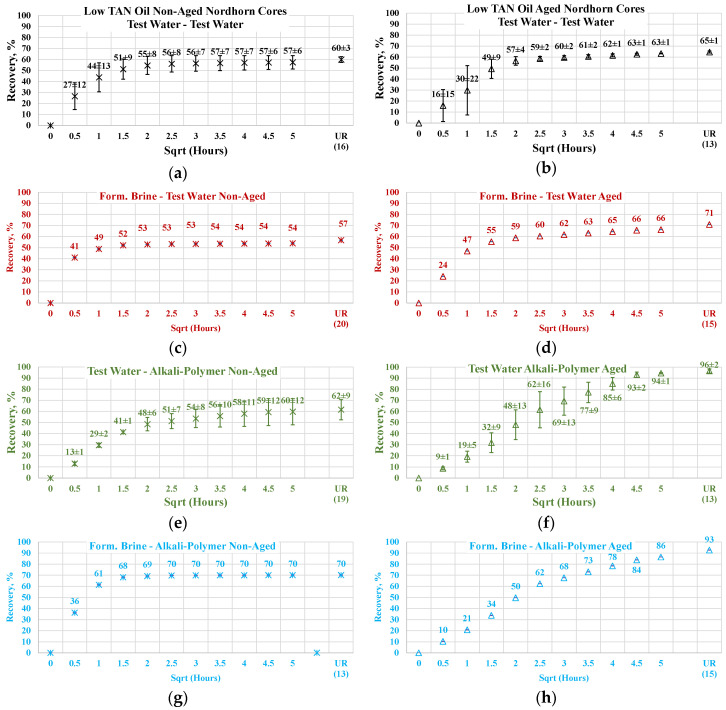
Percentage of production versus the square root of the time plot for non-aged and aged Nordhorn core plugs saturated with low TAN oil (water composition comparison) at 60 °C (alkali: 7 g/L Na_2_CO_3_; polymer: 2000 ppm FLOPAAM 3630S). Data of non-aged cores (**a**,**c**,**e**,**g**) are presented on the left side whereas aged core data (**b**,**d**,**f**,**h**) on the right side. Black-colored data refer to test water (saturated core) imbibed by test water; red-colored data refer to formation brine (saturated core) imbibed by test water; green-colored data refer to test water (saturated core) imbibed by alkali-polymer, and blue-colored data refer to formation brine (saturated core) imbibed by alkali-polymer. Ultimate recovery (UR) refers to recovered oil divided bv Soi.

The comparison of aged and non-aged Nordhorn core plugs is shown in Figure 18 for high TAN oil and in Figure 19 for low TAN oil. For the observations obtained in this work, when the core plug was placed into the test water after saturation, the composition of the formation brine did not play a large role. As can be seen from Figure 18a, the oil production profile followed the same path for both the formation brine and test water’s initial saturation in the case of aged and non-aged cores.

Differences could be detected when the alkali–polymer solution was used as displacing fluid. A higher production rate was visible when formation water was used for primary saturation. The observed behavior was in good agreement with the data reported by Haagh et al. [48], which can be explained by surfactant–divalent ion–rock surface interactions. Divalent ions can promote wettability alteration when used with anionic surfactants. When the alkali reacts with crude oil acids, an anionic surfactant with a negatively charged head is formed. The negatively charged end of this surfactant can create a bridge with the mineral surface. This effect yields faster wettability alteration, leading to a water-wet state and higher oil production rate. This statement is supported by the results of similar experiments performed with low TAN oil.

Comparing the obtained results of alkali-polymer imbibition for high and low TAN oil, one could notice that the production rate with high TAN oil is slower, which indicates that smaller capillary forces could be acting in the porous media. Smaller capillary forces occur due to smaller IFT thanks to the larger amount of anionic surfactants (soaps) created while reacting with high TAN oil. Ultimate oil recovery is also slightly higher in the case of high TAN oil compared to low TAN. These observations support the affirmation that application of alkali–polymer in high TAN oil leads to a higher degree of wettability alteration. The oil production profiles for low TAN oil are presented in Figure 19. As can be seen, the production behavior was similar for all cases, except for the non-aged alkali–polymer experiment. Notably, the same production profiles were observed among the aged cores with implementation of the alkali–polymer solution. In this case, the process of wettability alteration due to anionic surfactant creation did not occur. As a result, there are no observable differences between the saturation of core plugs with brine of high or low divalent ion concentration.

#### 3.3.4. Nordhorn and Keuper Core Plugs—Influence of Mineralogy

**Figure 20:**
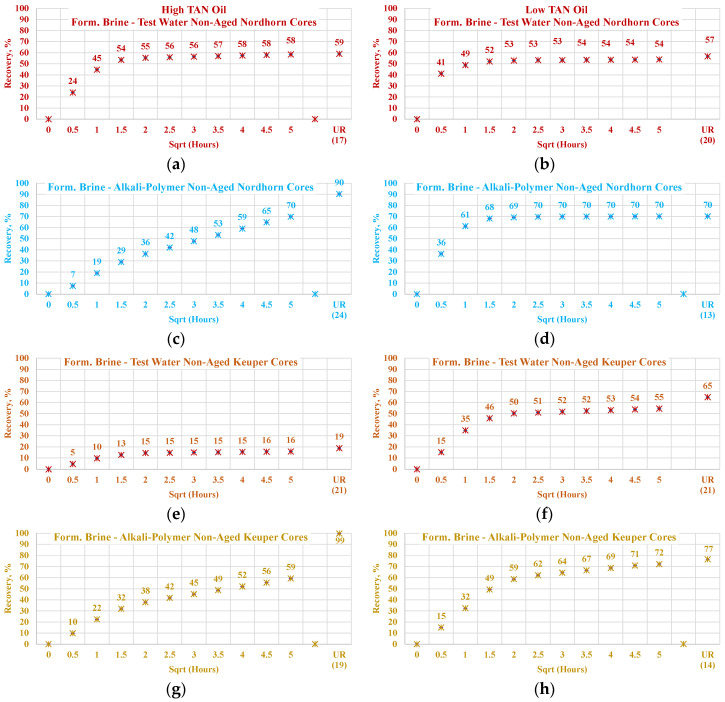
Percent of production versus square root of time plot for non-aged Nordhorn and Keuper core plugs for high TAN oil (**a**) and low TAN oil (**b**) at 60 °C (alkali: 7 g/L Na_2_CO_3_; polymer: 2000 ppm FLOPAAM 3630S). High TAN oil data (**a**,**c**,**e**,**g**) are presented on the left side whereas low TAN oil data (**b**,**d**,**f**,**h**) on the right side. Nordhorn cases are presented from (**a**) to (**d**), whereas Keuper cases are presented from (**e**) to (**h**). Red-colored data refer to formation brine (saturated core) imbibed by test water; blue-colored data refer to formation brine (saturated core) imbibed by alkali-polymer; orange-colored data refer to formation brine (saturated core) imbibed by test water, and mustard-colored data refer to formation brine (saturated core) imbibed by alkali–polymer. Ultimate recovery (UR) refers to recovered oil divided bv Soi.

For this set of experiments, core plugs were initially saturated with the formation brine containing a large number of divalent ions. After oil saturation, the cores were submerged into the test water and alkali–polymer solution. The observed oil production behavior is presented in Figure 20. The results indicate wettability alteration, even when the core was not aged. Therefore, the influence of TAN is clearly indicated. For high TAN oil (16 TH), the Keuper core plugs showed a significant decrease in both the production rate and ultimate oil recovery when test water was used as a displacing fluid. At the same time, oil production was similar to that of the Nordhorn core plugs in the case of alkali–polymer application. This suggests that even without the aging period, the polar components of the crude oil covered the clay mineral surfaces, resulting in a neutral (oil)-wet state of the core. However, during the application of the alkali–polymer solution, the oil-wetness returned to a water-wet state and, as a result, greater ultimate recovery was achieved. Similarly, small capillary forces led to slow production. The large difference in the case of test water application could also be related to the high value of heterogeneity in the Keuper outcrop core.

Wettability alteration was also indicated for the low TAN oil (St. Ulrich), with a slower production rate observed for the Keuper core plug. This demonstrates the neutral (oil)-wet state of the core after aging. The lower ultimate oil recovery for the Nordhorn core plugs can be explained by the “snap-off” effect in the water-wet system that leads to higher residual oil saturation. Another remarkable point is the minor improvement in oil recovery with the alkali–polymer solution. As demonstrated by IFT measurements, the low TAN oil failed to reduce the interfacial tension to low values. Vijapurapu and Rao [49] reported a critical value of IFT, above which wettability alteration does not occur [48]. When this critical IFT value is not reached, oil droplet detachment does not occur. Consequently, lower ultimate production is obtained.

**Figure 21:**
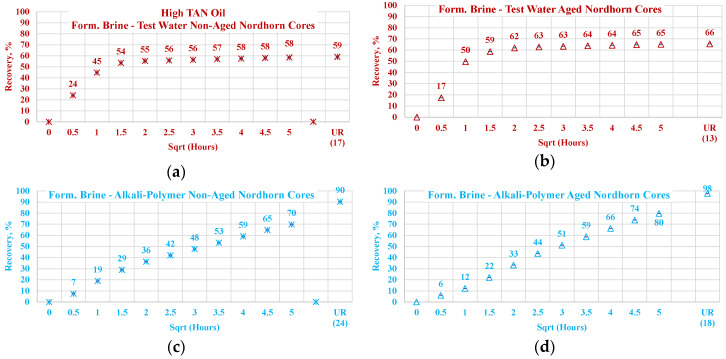
Percentage of production versus the square root of the time plot for Nordhorn core plugs (non-aged and aged), saturated with high TAN oil at 60 °C. Alkali: 7 g/L Na_2_CO_3_, Polymer: 2000 ppm FLOPAAM 3630Ss. Data of non-aged cores data (**a** and **c**) are presented on the left side, whereas aged cores data (**b** and **d**) on the right side. Red-colored data refer to formation brine (saturated core) imbibed by test water; blue-colored data refer to formation brine (saturated core) imbibed by alkali–polymer. Ultimate recovery (UR) refers to recovered oil divided bv Soi.

**Figure 22:**
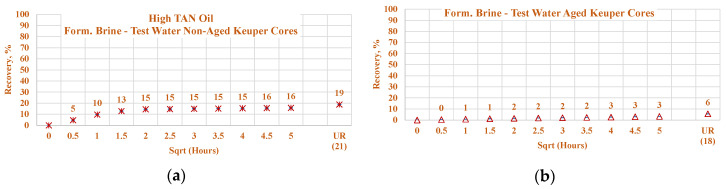

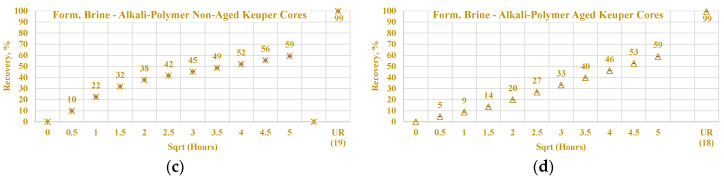
Percentage of production versus the square root of the time plot for Keuper core plugs (non-aged and aged), saturated with high TAN oil at 60 °C. Alkali: 7 g/L Na_2_CO_3_, Polymer: 2000 ppm FLOPAAM 3630Ss. Data of non-aged cores data (**a** and **c**) are presented on the left side, whereas aged cores data (**b** and **d**) on the right side. The orange-colored data refer to formation brine (saturated core) imbibed by test water and the mustard-colored data refer to formation brine (saturated core) imbibed by alkali–polymer. Ultimate recovery (UR) refers to recovered oil divided bv Soi.

**Figure 23:**
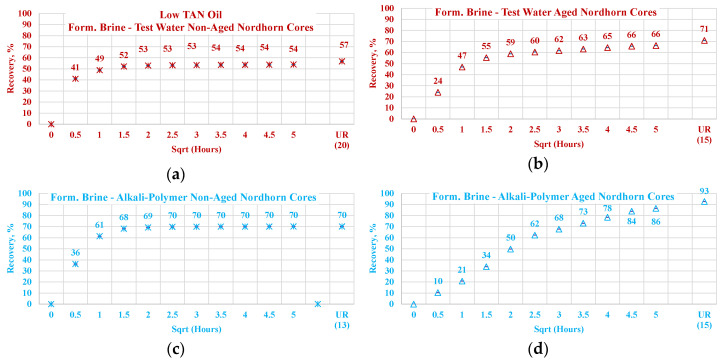
Percentage of production versus the square root of the time plot for Nordhorn core plugs (non-aged and aged), saturated with low TAN oil at 60 °C. Alkali: 7 g/L Na_2_CO_3_, Polymer: 2000 ppm FLOPAAM 3630Ss. Data of non-aged cores data (**a** and **c**) are presented on the left side, whereas aged cores data (**b** and **d**) on the right side. Red-colored data refer to formation brine (saturated core) imbibed by test water; blue-colored data refer to formation brine (saturated core) imbibed by alkali–polymer. Ultimate recovery (UR) refers to recovered oil divided bv Soi.

**Figure 24:**
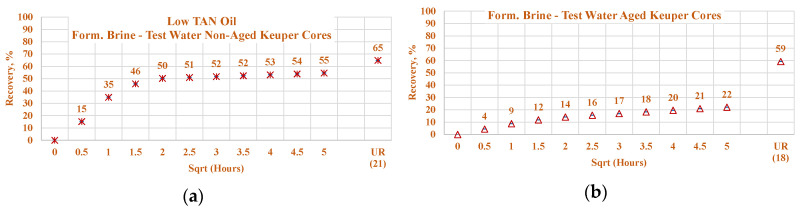

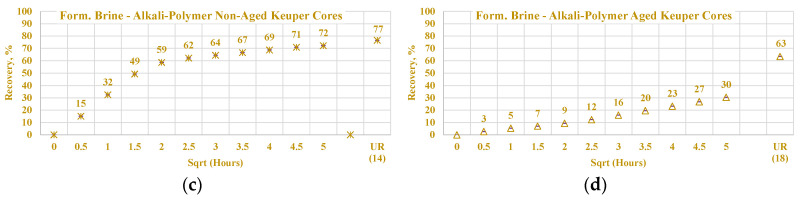
Percentage of production versus the square root of the time plot for Keuper core plugs (non-aged and aged), saturated with low TAN oil at 60 °C. Alkali: 7 g/L Na_2_CO_3_, Polymer: 2000 ppm FLOPAAM 3630Ss. Data of non-aged cores data (**a** and **c**) are presented on the left side, whereas aged cores data (**b** and **d**) on the right side. The orange-colored data refer to formation brine (saturated core) imbibed by test water and the mustard-colored data refer to formation brine (saturated core) imbibed by alkali-polymer. Ultimate recovery (UR) refers to recovered oil divided bv Soi.

Data of the experiments shown in Figure 21, Figure 22, Figure 23 and Figure 24 were used to assess the influence that aging can have on the Keuper outcrop during the spontaneous imbibition process. Figure 21 depicts the behavior of oil production from aged and non-aged Keuper samples and compares it to that of the Nordhorn samples (results previously discussed). Data obtained from the aged core plugs confirmed the wettability changes, and minimal oil production was observed for the aged Keuper core plugs using test water as a displacing fluid. This represents a significant wettability change towards an oil-wet state. Similarly, using the alkali–polymer solution, the oil production rate also dropped significantly, suggesting oil-wet behavior, but the ultimate oil recovery was the largest. This can be explained, as previously described, by the principle of the anionic surfactant–mineral surface reactions. Note that similar observations were obtained when evaluating the low TAN oil (see Figure 22). One important overall observation is that a significant shift in core wettability was observed for the Keuper outcrop. The observations were based mainly on the profile of the oil production plot. Both the aged Keuper core plugs showed much slower production rates and ultimate oil recovery, which are indicators of a strongly oil-wet system. The alkali–polymer application again failed to improve the oil recovery for low TAN oil.

## 4. Summary and Conclusions

The parameters and conditions defining the wettability alteration and IFT reduction in the alkali–polymer were identified for low and high TAN oils. The work showed how oil composition (low and high TAN), chemical type, brine composition, core plug condition (aged and non-aged), and reservoir mineralogy (2.5% and 10% clay content) clearly influence fluid–fluid and rock–fluid interactions. These results are used as a guidance to understand the field application envisaged in the Matzen 16 TH reservoir.

The interfacial tension between tested oils and test water (without divalent ions) remained constant. When alkali was added, the decrease in IFT for high TAN oil was more significant and reached the low equilibrium value. The addition of polymer to the alkali solution diminished the effect of IFT reduction and increased the observed IFT peak value.

Low and high TAN oil did not lead to a difference when using the test water. Regardless of the TAN of the oil, the imbibition rate was larger when using test water as a displacing fluid. For the polymer, this rate slowed, presumably due to the molecule size and viscosity of the solution. For the alkali and alkali–polymer, the slower imbibition can be explained by a reduction in capillary forces. For the non-aged core plugs, in the case of low TAN oil, all chemical solutions provided a similar ultimate recovery, while for high TAN oil, the use of alkali and alkali–polymer solutions led to a larger amount of produced oil.

Core aging (aged or non-aged) for the test water and polymer as displacing fluids reflected a slower production rate only within the first hour of the imbibition process. Other cases exhibited a significantly slower initial production rate. The lower ultimate oil production for non-aged (water-wet) core plugs can be explained by the oil droplet snap-off phenomenon. By submerging more oil-wet core plugs into different solutions, the invading fluid can enter the middle of the pore throat, displacing a larger amount of oil in the case of a water-wet core. Moreover, when alkali was used on the oil-wet core samples, the fluid imbibed larger pores and displaced the oil from the middle areas of the pore throats; then, the in situ-created surfactants caused wettability alteration. Invading fluid subsequently started to spread around the inner surface of the pore throats which led to oil detachment from the mineral surfaces. The residual oil saturation decreased for all aged Nordhorn core cases compared to non-aged samples with alkali and alkali–polymer.

The brine composition did not yield significant changes in the oil production rate or ultimate oil recovery when the formation brine or test water was used for initial saturation in the case of non-aged (water-wet) cores. For aged (neutral to oil wet) core plugs saturated with high TAN oil, a higher production rate was detected when the formation water was used for primary saturation and the alkali–polymer solution was used as a displacing fluid. For the low TAN oil, the divalent ions did not play a role in the case of aged cores with the implementation of the alkali–polymer solution.

According to the observed data, core mineralogy is linked to wettability alteration. Core samples containing 10% clay content exhibited neutral to oil-wet behavior even when the core was not aged. For high TAN oil, the Keuper (high clay content) core plugs showed a significant decrease in the production rate and ultimate oil recovery, indicating a strongly oil-wet system. During the application of the alkali–polymer solution with high TAN oil, the oil-wet sample returned to a water-wet state, and higher ultimate recovery was achieved. In the case of the low TAN oil, a minor improvement in oil recovery with the alkali–polymer solution was observed, meaning that no oil detachment from the mineral surfaces occurred. Overall, core plug mineralogy tremendously affects the process of wettability alteration due to aging and the implementation of chemical solutions; substantial wettability change effects were observed particularly when alkali was injected.

## Figures and Tables

**Table 1:** Overview of selected AP flooding evaluations reported in the literature.

No.	Chemicals	Experimental Approach	Recovery Mechanisms	Insightful Outcome	Reference
1	A/P/AP	Channeled sandpack flooding	Improved mobility ratio	Plugging and diverted flow	Wu et al. [8]
2	A/AP/ASP	Microfluidics chip	Viscosity of displacing fluid + pore-scale morphology	At same capillary number + mobility number	Alzahir et al. [9]
3	ASP	Daqing oilfield tests	Ultra-low interfacial tension (IFT) under oil-wet state	Weak alkali in ASP worked better than strong alkali in ASP	Sun et al. [10]
4	AP	Book chapter with field applications	Improved sweep efficiency + in situ soap generation	Low adsorption + better injectivity	Sheng.J. [11]
5	AP	Field projects + laboratory + simulation Summary	Polymer mobility control + soap improved displacement efficiency	Mixed outcomes from field tests	Sheng, J. [12]
6	AP	IFT study for Fang oilfield test	Lower IFT + improved mobility ratio	Effect of pressure, temperature, and salinity on IFT	Maneeintr et al. [13]
7	ACP	Sandpack slap flooding + simulation	Slug viscosity + ultra-low IFT	Optimum initiation time in heavy oil	Aitkulov et al. [14]
8	AP + Nanoparticles	Unsteady state displacement experiments	In situ soap generation, pressure increment	Higher oil relative permeability values	Mortazavi et al. [15]
9	ACP/AP	Micromodels, core flood	Improved sweep efficiency + in situ soap generation	Low adsorption, better sweep efficiency and cost efficiency	Schumi et al. [16]

A = alkali; P = polymer; S = surfactant; AP = alkali–polymer, ACP = alkali–cosolvent–polymer.

**Table 2:** Brine properties and composition used for the evaluations included in this work. Note that test water is the same formation of brine but softened to only NaCl, including a buffer for pH control of NaHCO_3_.

Parameter	Units	Test Water		Formation Brine	
		Mean	SD *	Mean	SD *
Density, 25 °C	Kg/m^3^	1011.700	0.000	1012.000	0.000
Density, 60 °C		997.100	0.100	1012.000	0.000
Shear Viscosity, 25 °C	mPa∙s	0.964	0.032	0.954	0.030
Shear Viscosity, 60 °C		0.571	0.023	0.592	0.054
Components	Units	Formation Brine		Test Water	
NaCl	g/L	19.750		18.960	
NaHCO_3_	g/L	-		1.850	
CaCl_2_·2H_2_O	g/L	0.400		-	
MgCl_2_·6H_2_O	g/L	0.660		-	
NH_4_Cl	g/L	0.170		-	
SrCl_2_·6H_2_O	g/L	0.060		-	
BaCl_2_·2H_2_O	g/L	0.030		-	

* SD—standard deviation.

**Table 3:** Properties of the oils used in this work.

Parameter	Units	16 TH		St. Ulrich	
		Mean	SD *	Mean	SD *
Density, 25 °C	Kg/m^3^	907.30	0.10	866.60	0.20
Density, 60 °C		884.30	0.20	842.60	0.60
Shear Viscosity, 60 °C	mPa∙s	11.90	0.10	6.00	0.10
TAN (total acid number)	mg KOH/g	1.61	0.18	0.17	0.08

* SD—standard deviation.

**Table 4:** Properties of the chemical solutions used in this work.

Parameter	Units	Alkali		Polymer		AP Solution	
		Mean	SD *	Mean	SD *	Mean	SD *
Density, 25 °C	Kg/cm^3^	1020.00	0.000	1013.00	0.00	1018.70	0.100
Density, 60 °C		1005.20	0.10	998.50	0.00	1004.20	0.000
Viscosity @7,94 s^−1^, 25 °C	mPa∙s	0.99	0.02	27.78	0.002	25.910	0.001
Viscosity @7,94 s^−1^, 60 °C		0.55	0.02	19.54	0.002	18.050	0.002
deviation							

* SD—standard.

**Table 5:** Overall core and saturation data for the outcrop samples used in this work.

Parameter	Units	Nordhorn		Keuper	
		Mean	SD	Mean	SD
Length	cm	8.010	0.110	8.120	0.090
Diameter		2.960	0.010	2.980	0.010
Bulk volume	cm^3^	54.420	0.870	55.760	0.730
Pore volume		13.070	0.260	12.750	0.220
Grain volume	kg/m^3^	41.360	0.700	42.980	0.670
Porosity	%	23.960	0.350	22.790	0.340
N_2_ permeability	mD	2313.000	162.000	1327.000	186.000
Water (test water) permeability		1500.000	190.000	902.000	198.000
Irreducible water saturation	%	25.600	4.000	32.500	4.400
Oil permeability	mD	314.000	79.000	178.000	51.000
End-point oil relative permeability	-	0.210	0.060	0.200	0.070
Initial oil saturation	%	74.400	4.000	67.500	4.400

**Table 6:** Contact angle results before aging the core plugs under various aqueous/oil conditions.

Core	Time (Min)	Contact Angle—16 TH			Contact Angle—St. Ulrich		
		TW *	Alkali	Alkali–Polymer	TW *	Alkali	Alkali–Polymer
Nordhorn	0	60.0	57.8	43.7	62.7	38.1	61.8
	300	58.7	57.2	57.8	60.7	42.7	59.8
Keuper	0	49.1	53.3	60.7	45.2	51.9	34.6
	300	50.4	54.2	56.1	50.0	47.8	55.9

TW * test water.
